# Complex Metacarpophalangeal Dislocation

**Published:** 2020-03-02

**Authors:** Minh Thu An, Joshua P. Kelley, Matthew P. Fahrenkopf, John P. Kelpin, Nicholas S. Adams, Viet Do

**Affiliations:** ^a^Michigan State University College of Human Medicine, Grand Rapids; ^b^Spectrum Health/Michigan State University Plastic Surgery Residency, Grand Rapids; ^c^Orthopedic Associates of Michigan, Grand Rapids

**Keywords:** metacarpophalangeal joint dislocation, complex dislocation, volar plate, index dislocation, open reduction

## DESCRIPTION

**Figure 1:**
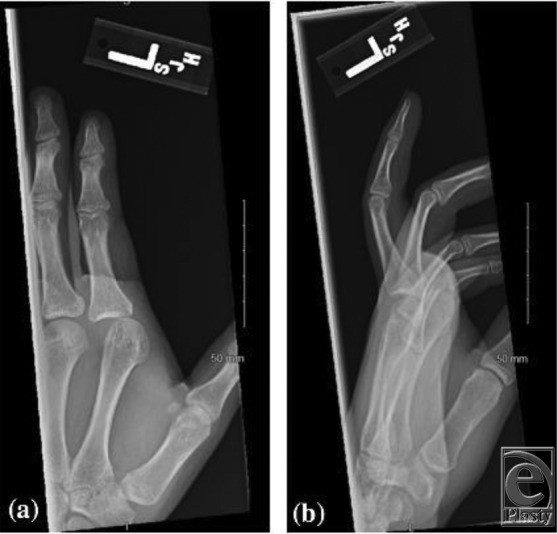
Posteroanterior (*a*) and lateral (*b*) radiographic images showing dorsal dislocation of the left index metacarpophalangeal joint and ulnar shift of the proximal phalanx.

A 16 year-old-male patient presented to the emergency department with a hyperextension injury at the left index finger, with resultant deformity of the metacarpophalangeal (MCP) joint. On examination, volar skin dimpling was noted and sensation was normal. Posteroanterior and lateral radiographs were obtained (Figs 1*a* and 1*b*). Closed reduction was unsuccessful, so the patient was taken to the operating room for open reduction.

## QUESTIONS

1. What are the causes of dorsal MCP dislocations?
2. What are the differences between simple and complex MCP dislocations?
3. What is the treatment of a complex MCP dislocation?
4. What are the complications and treatment outcomes?

## DISCUSSION

Dorsal MCP joint dislocations are infrequent injuries that are most commonly seen in the index and small fingers. These dislocations typically result from forced hyperextension of the digit on an outstretched hand.^1^ The resultant injury is a rupture in the membranous, proximal portion of the volar plate, joint capsule, and portions of the collateral ligaments. The volar plate can then become entrapped within the joint space between the base of the proximal phalanx and the dorsal metacarpal head.^1^

**Figure 2:**
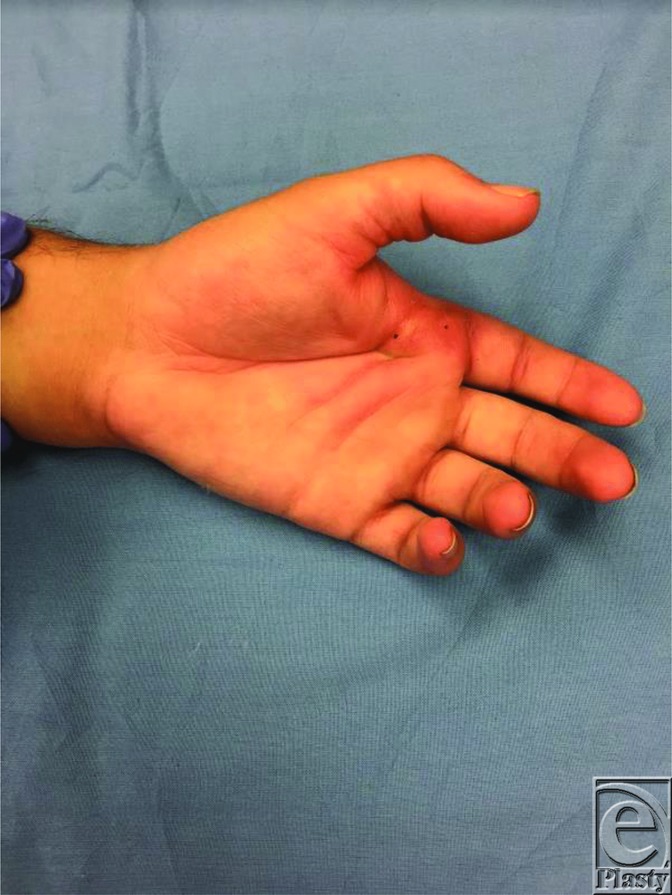
Hyperextension and volar skin dimpling are noted in this complex dislocation of the left index finger.

Simple dislocations are described as subluxations, where the volar plate remains in place over the metacarpal head.^2^ The collateral ligaments remain intact, allowing the joint to be easily reduced. The reduction maneuver for simple dislocations involve flexing the wrist to reduce tension on the flexor tendons and applying a distal, volar-directed force to the dorsal base of the proximal phalanx.^2^ In contrast, complex dislocations are not easily reducible, as the interposed volar plate or sesamoid bones precludes its reduction.^2^ Grossly, the MCP joint maintains a slightly extended posture. Dimpling of the volar skin is often seen (Fig 2). The flexor tendons can displace ulnarly, while the lumbricals displace radially to the metacarpal head. These structures can then tighten around the metacarpal neck, forming a noose-like structure, precluding closed reduction.^2^

**Figure 3:**
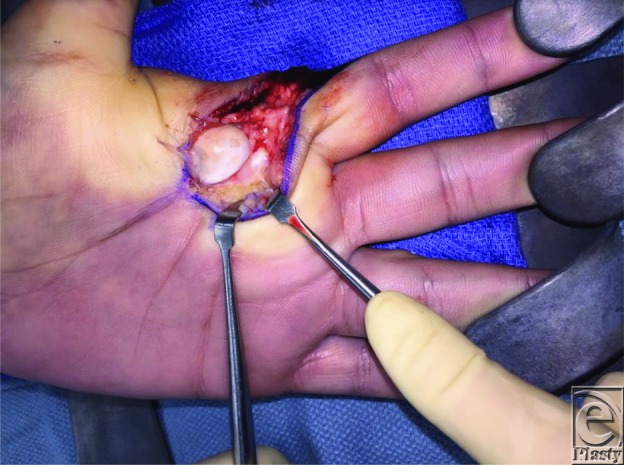
The dislocated metacarpophalangeal joint is visualized through a volar approach. The entrapped volar plate is seen adjacent to the metacarpal head.

**Figure 4:**
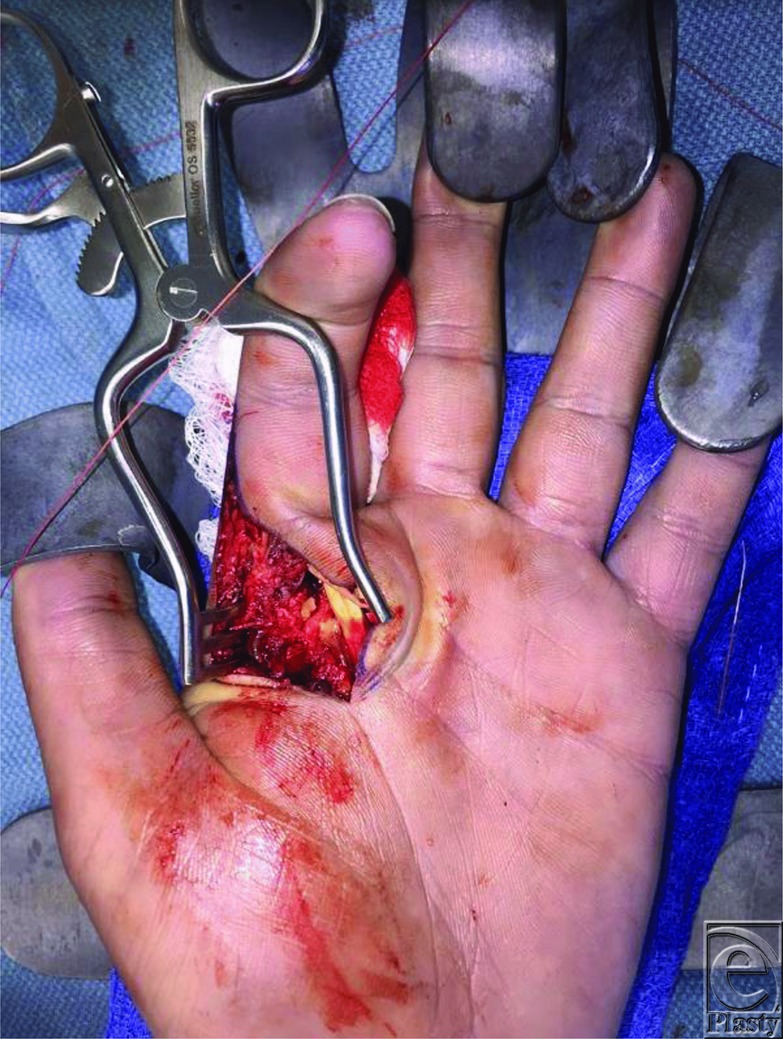
The tension from the A1 pulley was relieved to reposition the volar plate.

An open surgical approach is required with complex dislocations to remove interposed structures within the joint space and to release the “noose” around the metacarpal head. This can be performed through a dorsal or volar approach. The volar approach provides the best visualization of the entrapped volar plate (Fig 3). Advantages of using the volar approach include better access and visualization of the lesion, full repair of the volar plate, and lower risks of late instability by leaving the volar plate intact.^3^ The disadvantages include possible damage to the neurovascular bundle and difficulty accessing the volar plate because it is tented over the metacarpal head.^3^ Neurovascular damage can be prevented by meticulous dissection and careful handling of the digital nerves and vessels. For this procedure, a curvilinear incision was made over the MCP joint between the proximal and distal volar creases to expose the joint capsule. The tension produced by the muscle-tendon noose around the metacarpal neck was relieved by releasing the A1 pulley (Fig 4). By relieving the tension from the tendon, the proximal phalanx and the attached volar plate can be gently repositioned into the normal anatomic position.

Complications such as arthritis and osteonecrosis of the metacarpal head can arise from traumatic open reductions or late reductions.^2^ Damage to the neurovascular bundles is possible during open treatment.^3^ Excessive fibrosis and reduced final range of motion can result from prolonged immobilization or severe adjacent tissue injury.^3^ The best results are seen when operation is performed within the first day of injury. Postoperative management for isolated dorsal MCP joint dislocation includes dorsal splinting in a functional position up to 3 weeks.^4^ Full recovery of motion is commonly seen within 4 to 6 weeks.^5^

In summary, complex dorsal dislocations of the MCP joints are uncommon and are often treated with an open surgical approach. The volar surgical approach allows for better access and visualization of the strangulated metacarpal head and the volar plate. Careful dissection and handling of the neurovascular bundle are necessary to avoid damage to the digital nerves and vessels. If open treatment of irreducible MCP joint dislocations is performed in a timely manner, full recovery and range of motion are very likely within 4 to 6 weeks postoperatively.
